# Computational and molecular tools for scalable rAAV-mediated genome editing

**DOI:** 10.1093/nar/gku1286

**Published:** 2014-12-08

**Authors:** Ivaylo Stoimenov, Muhammad Akhtar Ali, Tatjana Pandzic, Tobias Sjöblom

**Affiliations:** Science For Life Laboratory, Department of Immunology, Genetics and Pathology, Rudbeck Laboratory, Uppsala University, SE-751 85 Uppsala, Sweden

## Abstract

The rapid discovery of potential driver mutations through large-scale mutational analyses of human cancers generates a need to characterize their cellular phenotypes. Among the techniques for genome editing, recombinant adeno-associated virus (rAAV)-mediated gene targeting is suited for knock-in of single nucleotide substitutions and to a lesser degree for gene knock-outs. However, the generation of gene targeting constructs and the targeting process is time-consuming and labor-intense. To facilitate rAAV-mediated gene targeting, we developed the first software and complementary automation-friendly vector tools to generate optimized targeting constructs for editing human protein encoding genes. By computational approaches, rAAV constructs for editing ∼71% of bases in protein-coding exons were designed. Similarly, ∼81% of genes were predicted to be targetable by rAAV-mediated knock-out. A Gateway-based cloning system for facile generation of rAAV constructs suitable for robotic automation was developed and used in successful generation of targeting constructs. Together, these tools enable automated rAAV targeting construct design, generation as well as enrichment and expansion of targeted cells with desired integrations.

## INTRODUCTION

Targeted engineering of the human genome in somatic cells is a powerful means to study functional consequences of mutations found in the genomes of cancer cells or in patients with inherited genetic disorders, and potentially also for gene therapy of these diseases. One class of such tools, encompassing the zinc finger nucleases (ZFNs), Transcription Activator-Like Effector Nucleases (TALENs), homing endonucleases, triplex-forming oligonucleotides and Targetrons, are engineered molecular scissors that enable targeted genome editing at high efficiency (1–4). In mammals, the site-specific DNA double-strand breaks (DSBs) created by the nuclease domains of these enzymes trigger DNA DSB repair and result in >1% desired targeting events (1,3). The ZFNs are customizable and well characterized in terms of specificity, affinity and genotoxicity, but have a bias toward G-rich sequences. However, frequent mutations because of non-homologous end-joining (NHEJ) repair and off-target cleavage at sites not predicted *in silico* are key issues (3,5–9). As ZFNs have to be engineered separately for every targeted site, they are expensive and require expertise to design. However, several open-access platforms are likely to transform the use of ZFNs and TALENs in the future (10). The recently developed Cas9/CRISPR system allows gene targeting guided by RNA, and may be particularly useful for gene knock-out although the targeting specificity remains to be determined (11,12). The CRISPR targeting efficiency is up to 25% in human somatic cells and multiplex human genome editing has been performed as well as forward functional genomic screens (13,14). In spite of high efficiency and versatility, the specificity remains a limitation to generate true isogenic cell lines using these molecular scissors. A recent whole genome sequencing study of CRISPR- and TALENs-based gene targeting in human cells revealed off-target mutagenesis ranging from small indels and single-nucleotide variants to structural variants. Further, none of the detected indels were within predicted potential off-target sequence while allowing up to six mismatches (15). The off-target related mutagenesis in CRISPR-based technologies can be partially addressed by the use of Cas9 nickase mutants in combination with paired guide RNAs (16). Collectively, these technologies constitute efficient tools for genome editing but may give rise to off-target editing.

Adeno-associated virus (AAV) vectors constitute a well-established means to edit the genome of human somatic cells by homologous recombination (HR) (17). The AAV2 virus has a single-stranded DNA genome with a packaging capacity of 4.7 kb, can integrate in dividing and non-dividing cells and has a gene targeting efficiency from 10^−5^ to 10^−2^(18). The targeting efficiency can be enhanced up to 0.12% in human pluripotent cells by directed evolution of the AAV vectors (18–21). The rAAV targeting vectors can be constructed either by conventional cloning, 3-way fusion polymerase chain reaction (PCR), or 3-way ligation (17,18,22). While providing a faster route to final construct than conventional cloning, the latter approaches may be less well suited for large-scale generation of rAAV constructs as the experimental conditions need to be optimized for each targeting construct. The rAAV method requires extensive human effort to design constructs to achieve mono-allelic knock-in or bi-allelic knock-out, usually in several distinct steps. A computationally assisted approach could accelerate and standardize the highly repetitive tasks of selecting homology arms (HAs) and designing intermediate components such PCR primers. Such an approach would have to adhere to the empirically known design criteria: (i) ≤25% repeat content in homology arms, (ii) short distance between HAs for better integration efficiency, (iii) homology arms designed for facile PCR amplification, (iv) for knock-in designs, exon:intron boundaries must be preserved to not affect splicing, any scars caused by vector integration must not affect any other exons of the gene and the integration site should be as proximal as possible to the target exon for efficient HR at a target base inside the exon as the probability of retention of the knock-in modification decreases with the increasing distance from the integration site (23), (v) for complete gene knock-out, targeting should be restricted to exons present in all transcript variants of the gene and whose length is non-divisible by 3 to eliminate the risk of exon skipping which may produce functional or hypomorphic protein products.

Here we provide (i) a database of strategies for knock-out or knock-in for the majority of bases and exons in human protein encoding genes, (ii) a vector family and protocol for rAAV gene targeting in human somatic cells, encompassing a rapid and efficient Gateway approach to generate the targeting construct, universal screening PCR primers with internal controls which serve as a template positive and event negative control for both the construct making and locus-specific integration and cell surface markers for sorting of cells with genomic integration of the targeting construct.

## MATERIALS AND METHODS

### Databases of exons in human protein-encoding genes

The consensus coding DNA sequence definitions of human protein coding regions and exon coordinates were downloaded from the ftp server of the CCDS project (CCDS, Release 15 on 29 November 2013, ftp://ftp.ncbi.nlm.nih.gov/pub/CCDS/archive/15/CCDS.20131129.txt). The file CCDS.20131129.txt was parsed, records with CCDS status ‘Public’ were kept and each transcript was assigned a CCDS identifier and GeneID. Exons sharing the same GeneID and same genomic coordinates, but having a different CCDS identifier (i.e. being found in alternative transcripts of the same gene) were compressed to one entry, while keeping track of the different CCDS identifiers. A subset of exons shared the same GeneID and different, but overlapping genome coordinates. We superimposed all coordinates and kept the outermost coordinates to create an entity termed ‘exon projection’ representing the borders within which several different exons from transcript variants of the same gene were found. Following the compression of redundant exons and the creation of exon projections, genomic sequences with 3 Kb flanking sequences from the 3′ and 5′ direction were extracted from the human reference assembly. Relevant information on exons and exon projections, including GeneID, CCDS track record, chromosome number, genome coordinates, strand orientation, sequence including the flanking 3 Kb and in case of different transcript variants a record of how common every exon or exon projection is in all known transcript variants was stored in a SQLite database (ExonProjectionsDB.sqlite) and used for computation of gene knock-in scenarios. A similar database (ExonDB.sqlite), containing all public exons without compression or exon projections was created for generation of gene knock-out scenarios.

### Optimization of homology arm design

The computation time, the total number of stored primer pairs and the sequence coverage are all influenced by three main parameters: the size of the sliding window (SW), the step size (SS) and the number of top scoring primer pairs (NPP) stored after each round of primer design. SW was fixed to 1300 bases (>1 *HA*_max_length_, < 2 *HA*_min_length_). To find practical values of SS, NPP and overall computational time, we compared the databases of HAs generated in CCDS exon projections and their flanking sequences on chromosome 21 while varying the parameters SS and NPP. The sequence coverage was defined as the number of nucleotides in the regions of interest present in at least one generated homology arm. The average sequence coverage was defined as the mean value of the number of times a specific base in the target region was covered in a homology arm. The average penalty value is the mean of PRIMER_PAIR_0_PENALTY scores calculated by Primer3 for each primer pair in the set.

### Databases of homology arms for CCDS genes

For each public GeneID in the CCDS database, a separate SQLite database file was created to store all potential PCR primer pairs associated with the exons of that GeneID. PCR primer design was performed using Primer3 release 2.3.5 (24) in a SW approach over a sequence of interest with several simultaneous Primer3 instances. Each Primer3 instance was forced to produce a PCR product in a different size range for each visible sequence window of 1300 bp. The size ranges were from 700 to 1200 bp in 50 bp length increments. The 50 primer pairs ranked highest by Primer3 (10 times the default value) for each size range for each window were collected and filtered. The filtering criteria were (i) no nucleotide of the PCR primers should reside in genomic repeats (implemented by providing Primer3 with repeat-masked sequence and PRIMER_MAX_NS_ACCEPTED = 0), (ii) mononucleotide runs in PCR primers were limited to 3 bases, (iii) the product of a suitable primer pair (homology arm) should not contain more than 25% sequence from genome repeats. The PCR primer length was 18–30 bp and all other parameters of Primer3 were default values.

### Generation of rAAV knock-in vector designs

To create targeting vectors for each exon or exon projection, we accessed the full set of HAs from the respective gene database, but limited the reach to a window of the target exon length plus 3 Kb in both the 3′ and 5′ directions. For each exon or exon projection we identified all HAs which spanned the whole exon and end at least 20 bases outside the exon borders. If at least one such exon spanning arm existed and if there were additional arms within 700 bases from either end of the spanning arm, we aimed to generate all possible arm pairs. There were thus two groups of scenarios, left-arm:spanning-arm (LS) and spanning-arm:right-arm (SR). We next performed clustering analysis of all spanning arms and the arms in 3′ direction (left) and 5′ direction (right) by the Density-Based Spatial Clustering of Application with Noise (DBSCAN) algorithm. Once the clusters of arms in each category (spanning, left and right) were defined we matched all arms from each spanning arm's cluster to all arms of the left and/or right arm clusters. Only the top scoring scenarios in each cluster-cluster matching were processed further. The scoring criteria were minimum gap size between the arms and proximity of the split-point to the exon borders. The selection criteria to score and rank the best cluster-cluster HA matches included priority for smaller gap between HAs, shorter distance from the split-point to the exon-intron border of the span arm and longer cumulative HA length. Scenarios which included other exons inside the gap between the arms or where the arms were ending in other exons were excluded. All top scoring scenarios from cluster-cluster matching were collected separately for each of the two principal groups (LS and RS) and compared inside a group toward each other to rank and select the best scenarios. The selection was based on a priority over smaller gap between the arms, smaller distance between the split point and the exon borders and maximizing the cumulative length of both homology arms. Scenarios which shared identical homology arms or highly similar arms were grouped as undirected graph and only the top scoring scenario in each graph was processed further. After the ranking of all scenarios in each of the two main groups (LS and RS) maximum 5 in each category were stored in a separate SQLite database for a given gene. A graphical output with all suggested knock-in scenarios for each gene was generated using the Python modules Image and ImageDraw.

### Generation of rAAV knock-out vector designs

The design of HAs for gene knock-out was similar to the knock-in approach above, with a few exceptions. All protein coding exons without any compression or exon projection were used. For each gene we then selected exons which were present in all alternative transcript forms and had a length not evenly divisible by 3. For every such exon we attempted to find all HAs which end in the exon (left HA) and HAs which start in the exon (right HA) to achieve a construct with split point inside the exon. If such arms existed, we performed DBSCAN clustering in each group. After defining the clusters of left HAs and right HAs, we attempted cluster-cluster matching with all HAs in each left cluster to all HAs in each right cluster. For every cluster-cluster matching we selected the one design having the smallest gap between HAs. These were collected and scored toward each other by minimizing the gap between the HAs and maximizing the cumulative length of HAs. Similar scenarios were grouped in an undirectional graph and only the best scoring scenario in each graph was processed further. Up to 5 such ranked scenarios per exon were stored in the final database. If the length of the exon in question was ≤700 bases, we also attempted to find whole exon excision designs by selecting left HAs ending in >20 bases from the exon start and right HAs starting >20 bases after the exon end. The algorithm for selection of the best five designs for whole exon deletion was identical to the one described above. The scenarios were collected in separate SQLite database files for each gene. A graphical summary of the selected designs was also created for each gene.

### Plasmid construction

Primers used in construct generation are listed in Supplementary Table S1. The PCR conditions were initial denaturation at 98°C for 3 min, three cycles of denaturation at 98°C for 20 s, annealing at 64°C for 20 s and extension at 72°C for 30 s per kb of amplicon followed by three cycles at 61°C and 58°C annealing temperature, respectively. The final amplification had 25 cycles of denaturation at 98°C for 20 s, annealing at 57°C for 20 s and extension at 72°C for 30 s per kb. The pAAV-Dest Gateway destination vector was assembled by cloning a fragment from the pHGWA (25) plasmid containing *att*R1, chloramphenicol resistance gene, the *ccd*B gene and *att*R2 between the *Not*I sites of the pAAV-multiple cloning site (MCS) vector (Stratagene). A fusion PCR approach was used where two fragments from the pHGWA insert were amplified using primers 1–4 such that the *Not*I site was removed.

### Generation of transmembrane fusion resistance genes

The pDisplay vector (Invitrogen) was used as a template for amplification of a fragment containing Ig K-chain leader sequence, hemagglutinin A (HA-A) epitope, myc epitope and platelet derived growth factor receptor (PDGFR) transmembrane domain. Prior to PCR amplification a single base was inserted into the MCS located between HA-A and Myc by site-directed mutagenesis (Stratagene) to preserve the reading frame of downstream element (PDGFR). Two fragments, one encompassing a part of the left *loxP* site together with internal ribosome entry site (IRES) and the other spanning the neomycin resistance gene and a part of the right loxP site were amplified from the pSEPT gene targeting plasmid (26). All three fragments were first amplified independently using primers 5–10 with overlapping ends by using Phusion DNA polymerase (Finnzyme) and then assembled by fusion PCR. The primers used in the final fusion PCR were tailed by *att*B4r and *att*B3r sequences and the fragments were recombined with the MultiSite Gateway plasmid pDONR P4r-P3r to generate entry clones with sorting vectors.

### Construction of promoterless gene targeting constructs

To engineer green fluorescent protein (GFP) containing versions of pBUOY2 with different antibiotic resistance genes (blasticidin, hygromycin, neomycin, puromycin and zeomycin), the GFP sequence was amplified from hpGK-GFP using Phusion DNA polymerase (Finnzyme) and tailed with *Pst*I restriction sites using primers 11 and 12. The amplified PCR product was cloned in mutated pDisplay by using *Pst*I DNA restriction enzyme (Fermentas) to generate the pDisplay-GFP vector. Then the following DNA fragments were PCR amplified by using phusion primers 13–34 from their respective vectors: (i) IRES fragment from pBUOY2 with 5′ *loxP* sequence and 3′ overlapping sequence to IgK-chain leader, (ii) IgK-chain leader-HA-GFP-Myc-PDGFR from pDisplay-GFP, with overlapping sequence to fragment 1 on the 5′ end and to their respective antibiotic resistance genes on the 3′ end, (iii) antibiotic resistance gene sequences with 5′ and 3′ overlapping sequence to fragments 2 and 4, respectively, and (iv) pA-loxP with 5′ overlapping sequence to fragment 3 and on 3′-end with partial *att*B4r sequence. These fragments were then assembled by fusion PCR in their numeric order for all five antibiotic genes and the final rounds of amplifications in each case were carried out with primers 5 and 10 tailed with *att*B3r and *att*B4r. These fragments were then recombined with MultiSite Gateway plasmid pDONR P4r-P3r vector to generate final entry clones. These entry vectors were termed pIRES.GFP.X where X is gene symbol of antibiotic resistance genes in it, e.g. pIRES.GFP.Bsd.

### Construction of promoter-containing gene targeting constructs

To increase the expression level of the sortable epitope, independent of target locus, promoter-containing versions of pIRES.GFP.X vectors were constructed by replacing the IRES sequence with an SV40 promoter. First, an SV40 promoter sequence with 5′ *loxP* and 3′ overlapping sequence to IgK-chain leader sequence was amplified from the p5A vector by using primers 35 and 36. Next, an IgK-chain leader-HA-GFP-Myc-PDGFR-X-pA with 5′ overlapping sequence to the SV40 fragment was amplified with primers 10 and 37 from the respective pIRES.GFP.X vectors and the fragments were fused by fusion PCR using *att*B3r and *att*B4r tailed primers 5 and 10. The respective products were then recombined with the MultiSite Gateway plasmid pDONR P4r-P3r vector to generate final entry clones. These entry clone vectors were termed pSV40.GFP.X, e.g. pSV40.GFP.Bsd. To further increase the ratio between targeted integrations and random integrations, the pSV40.GFP.X.pA vectors were further modified by replacing the polyA sequences with a DNA fragment containing foot and mouth disease virus 2A self-cleaving peptide and adenoviral splice donor sequence (FMVD2A.SD). In a first step, fragments containing X-overhang.FMVD2A.SD.loxP-(partial) were amplified using primers 38–43. In a second step, these fragments were used as template along with their respective pSV40.GFP.X vectors, to amplify a fused fragment containing attB4r.loxP.IgK-chain leader-HA-GFP-Myc-PDGFR-X-FMVD2A.SD.loxP-partial-attB3r, by using primers 5 and 44. In the final step, attB4r.loxP.IgK-chain leader-HA-GFP-Myc-PDGFR-X-FMVD2A.SD.loxP-attB3r fragment was amplified with primers 5 and 10 using purified PCR product from second step as template. The product was recombined with the MultiSite Gateway plasmid pDONR P4r-P3r vector to generate the final pSV40.GFP.X.SD entry clones.

### Assembly of gene targeting constructs

To obtain the gene targeting constructs, homology arms with their respective *att*B recombination sites were amplified with att*B* tailed primers using Platinum *Taq* high fidelity DNA polymerase (Invitrogen) from genomic DNA of HCT116 cells (Supplementary Table S1). The PCR conditions were initial denaturation at 96°C for 3 min, 3 cycles of denaturation at 96°C for 20 s, annealing at 64°C for 20 s and extension at 72°C for 60 s per kb of amplicon followed by three cycles at 61°C and 58°C annealing temperature, respectively. The final amplification had 25 cycles of denaturation at 96°C for 20 s, annealing at 57°C for 20 s and extension at 72°C for 60 s per kb. Next, 100 ng each of HA1 and HA2 PCR products were recombined with 150 ng of pDONR^TM^ P1-P2 and pDONR^TM^ P3-P4, respectively, using BP Clonase II (Invitrogen, 11789–020) according to the manufacturer's instructions. The resulting entry clones were screened for the presence of HAs by colony PCR amplification using Platinum *Taq* DNA polymerase (Invitrogen) and M13 priming sites 45–46 flanking the cloned HAs in the pDONR vectors. When necessary, knock-in mutations were introduced by site-directed mutagenesis (Stratagene) in the pEntry-HA vectors. Next, 10 fmol of each of pEntry-HA1 vector, the entry clone encoding the fusion resistance gene, and pEntry-HA2 vector were recombined with 15 fmol of pAAV-Dest vector using LR Clonase II (Invitrogen) according to the manufacturer's instructions. The correct orientation of all the three components in the final targeting construct was confirmed by colony PCR using LR screening primers 47–50.

### Generation of rAAV particles

Virus production and infection was performed as described (17). The AAV293 packaging cell line (Stratagene) was maintained in Dulbecco's modified Eagle's medium and the HCT116 colorectal cancer cell line in McCoy's 5A medium at 37°C and 5% CO_2_. Media were supplemented with 10% fetal bovine serum and 1% penicillin-streptomycin (Invitrogen). To produce rAAV particles containing single-stranded targeting DNA, 5 μg of each targeting construct, pHelper and pRC (Stratagene) were co-transfected into 80% confluent AAV293 cells in a 75 cm^2^ flask using Lipofectamine 2000 (Invitrogen). The rAAV particles containing the targeting construct were harvested as crude cellular lysate 48 h after transfection.

### Enrichment of cells with rAAV integration

The rAAV containing lysates were used to infect 5–6 × 10^6^ HCT116 cells seeded 24 h before. Twenty-four hours after infection, the medium was replaced by selection medium containing 8 μg/ml of blasticidin or 450 μg/ml of G-418 and clones with AAV integrations were selected for 2–3 weeks. The cells were lysed using Lyse-N-Go (ThermoScientific) and screened for site-specific integration by PCR.

### Fusion protein expression analysis

Cells were seeded in LabTekII 8 well chamber slides (Nunc) and allowed to attach overnight. As a positive control, cells transiently transfected with a modified pDisplay vector were used. Transfection was done with Lipofectamine 2000 (Invitrogen) accorning to manufacturer's instructions. Cells were fixed in 3.7% formaldehyde (Sigma-Aldrich) for 15 min at room temperature and permeabilized with 0.1% Triton X-100/phosphate buffered saline (PBS) for 10 min at room temperature. After blocking in 3% bovine serum albumin (BSA)/PBS for 40 min at room temperature, cells were incubated with anti-Myc mAb (71D10, Cell Signaling; 1:200) diluted in 3% BSA/PBS for 16 h at 4°C. The slides were washed and incubated with Alexa Fluor 555 donkey anti-rabbit (Invitrogen, 1:1000) secondary antibody for 1 h at room temperature. Cell nuclei were stained with Hoechst 33342 (Invitrogen, 1:10 000) for 40 min and images were taken with a Zeiss AxioImager M2 fluorescence microscope.

## RESULTS

First, we generated, ranked and selected potential knock-out and knock-in scenarios for all exons in all human genes that are eligible to be edited by rAAV technologies. Next, we generated suitable homology arm amplification primers for construction of rAAV vectors. Finally, we provide and validate a Gateway vector system for high-throughput generation of rAAV constructs.

### Databases of human exons

The basis for generation of homology arms was a SQLite database containing the most conservative and curated set of genes and their respective exon definitions publicly available, i.e. the CCDS project. In CCDS Release 15 there were 29 008 public transcripts from 18 667 genes with a GeneID, together having 305 464 exons. We omitted transcripts which were not public by CCDS definitions, e.g. those without GeneID, known pseudo-genes, putative genes and genes under review. To handle different transcript variants of a gene, we introduced the concept of exon projections (see Materials and Methods). Exons from all transcription forms of a gene were compressed into a single entry and exon projections were created for all exons sharing the same gene but having different but overlapping genome coordinates, thereby obtaining 188 900 unique exons and exon projections covering a total sequence length of 32 533 289 bases. The length of exons and exon projections ranged from 1 to 21 693 bases (median 123 bases). Of the exons or exon projections in the database, 97.3% were <700 bases. The database was then used to find suitable gene knock-in scenarios. To compute knock-out scenarios, a second SQLite database containing all exons and genes with no compression or exon projections was created. The sequence length of the exons in this second database was also 1–21 693 bases (median 122 bases).

### Optimization of homology arm design

**Figure 1.**
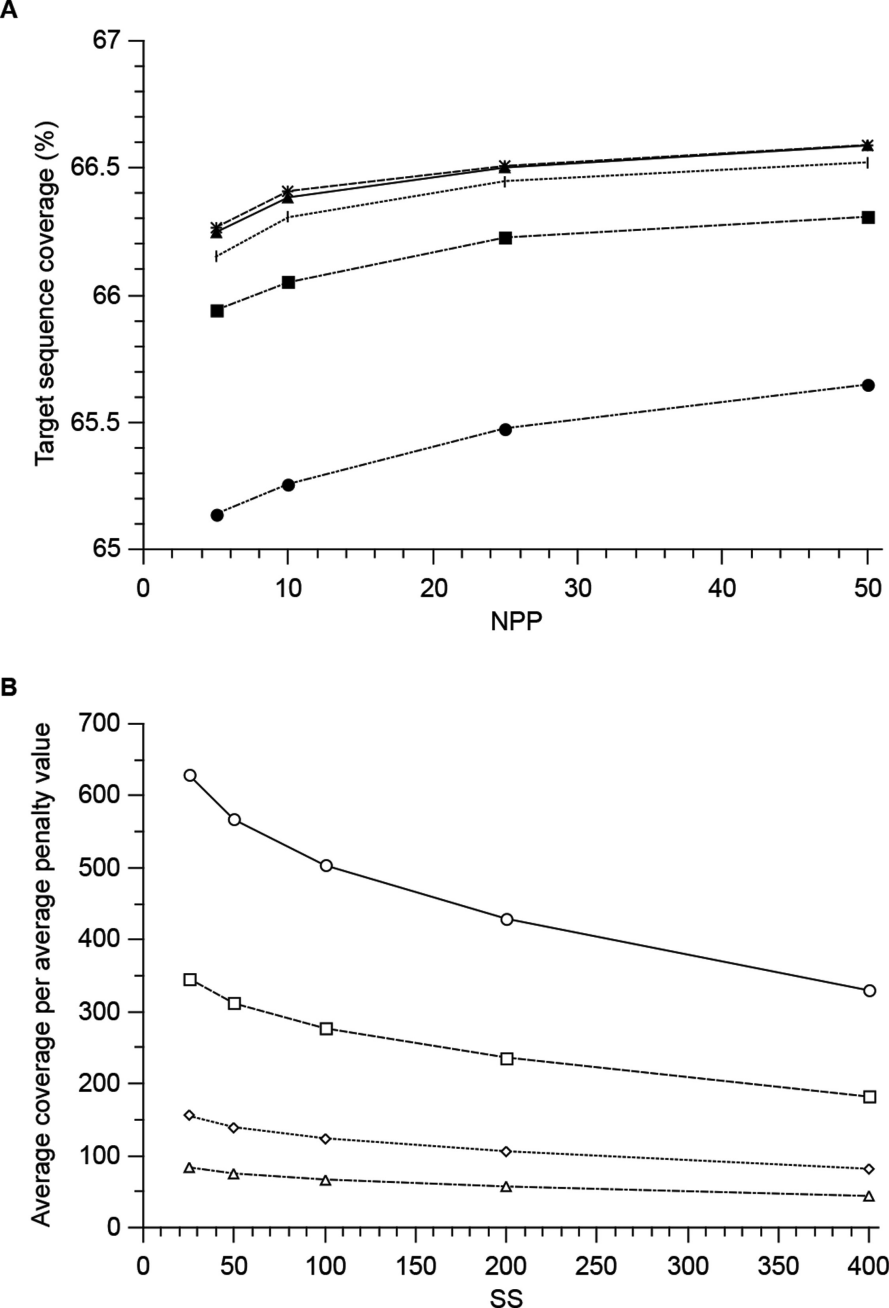
Optimization of homology arm design parameters. The sequence coverage (A) and the average coverage per average penalty (B) of coding exons from the 207 CCDS genes on chromosome 21 as a function the SS and the number of top scoring primer pairs (NPP) stored after each round of primer design. The SW size was kept constant at 1300 nt. (**A**) Fraction of the total target sequence covered by at least one PCR product as a function of NPP at different SS: 25 (*), 50 (▴), 100 (|), 200 (▪), 400 (•) nucleotides. (**B**) The average sequence coverage per average penalty as a function of SS at different NPP: 5 (Δ), 10 (⋄), 25 (□), 50 (○) retained primer pairs.

The generation of homology arms is the time limiting step in the pipeline, influencing sequence coverage, size of the HA database and the number of potential targeting scenarios. The time spent in this step is dependent on the size of the SW, the SS and the number of top scoring primer pairs (NPP) stored after each round of primer design. For SW, a value of 1300 was chosen to allow primer design freedom for products with maximum HA size, but restrictive to redundancy for generation of smaller products. To find practical values of SS and NPP, we compared the resulting sequence coverage of generated homology arms generated to 207 CCDS genes on chromosome 21 (Figure 1). The smaller the SS, the better sequence coverage and possibilities for PCR primer design, but the more redundancy and computational effort. Similarly, larger NPP gave more choice for potential homology arms through an increase in the average sequence coverage at the expense of increased computation time and database size. The change in sequence coverage was within ∼2% with the variation of both SS and NPP (Figure 1A). On the other hand, there was an increase in the depth of HA coverage (average sequence coverage) with decreasing SS and particularly with increasing NPP. The average penalty value increases when more primer pairs are demanded from Primer3. However, the average sequence coverage per average penalty value, which indicates the primer quality at a mean coverage depth, was more favourable at high NPP (Figure 1B). Since the availability of potential homology arms for any genome position is dependent of the average sequence coverage, we sought to maximize NPP, but also to minimize SS and selected values of SS = 50 and NPP = 50. In the sample set the average availability of HA for a genome position was >2000 for the chosen values of NPP and SS, with total sequence coverage close to the maximum. Also, the average coverage per average penalty value was second best at the chosen conditions, but with twice as fast computation time as the best condition.

### Databases of homology arms for each gene

Genome engineering by rAAV-mediated HR requires two homology arms, each of 700–1200 bp, surrounding the desired alteration. In practice, the HAs are often generated by PCR amplification from a template genomic DNA and we therefore sought to generate all potentially suitable PCR products of size 700–1200 bp for every exon and exon projection. We used a SW through the coding exon sequences and flanking 3 kb in 3′ and 5′ directions to supply Primer3 with input sequence for primer generation. All unique PCR primer pairs which fulfilled the design criteria were considered potential HAs and were retained, while redundant primer pairs were discarded. For the 18 667 attempted genes, we generated >7.09 × 10^8^ potential HAs with ≥1 HA in 99.4% of attempted genes (Supplementary Table S2). The average number of potential HAs per gene was 37 984 with an average of 3754 HAs per exon or exon projection. There were no suitable primer pairs fulfilling the selected criteria for 12 of the genes (0.06%) and these genes were excluded from further consideration.

### Generation of knock-in scenarios for protein-encoding genes

**Figure 2.**
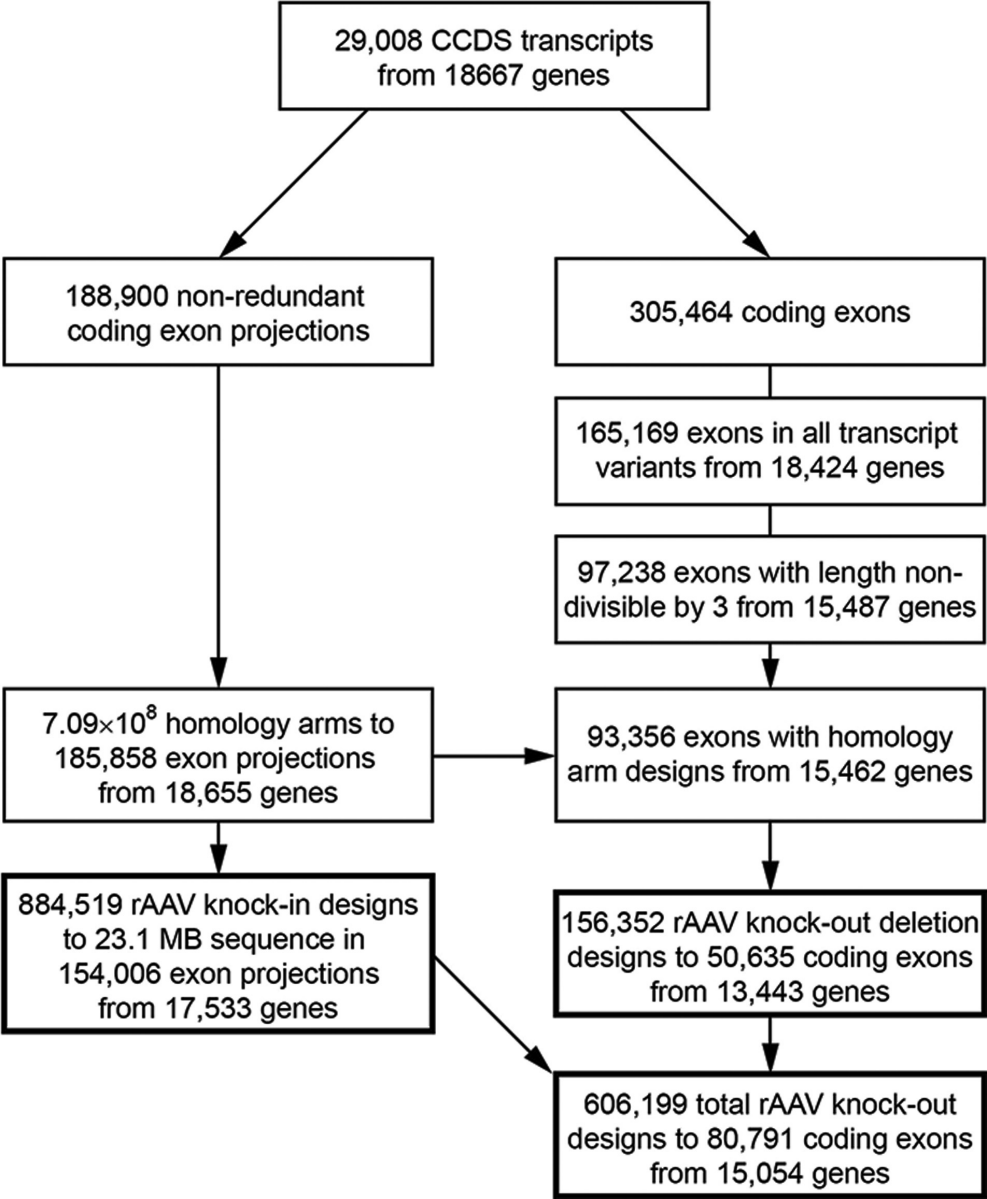
*In silico* design of rAAV gene targeting constructs to the compendium of human protein encoding genes. For knock-in designs, the protein encoding exons of different transcript variants of each gene were projected to obtain the outer boundaries of each coding feature and avoid vector integrations in splice junctions. Exon projections and their flanking sequences were used to design primers in defined product size ranges by a SW approach to generate a database of potential homology arms in the region. The most ten of the best ranked gene targeting construct designs per exon were then selected from the homology arm database. For knock-out, the design effort was restricted to exons present in all known transcript variants of a gene with exon length non-divisible by 3. Whole and partial exon deletion designs as well as stop codon insertion designs based on knock-in scenarios were generated.

**Figure 3.**
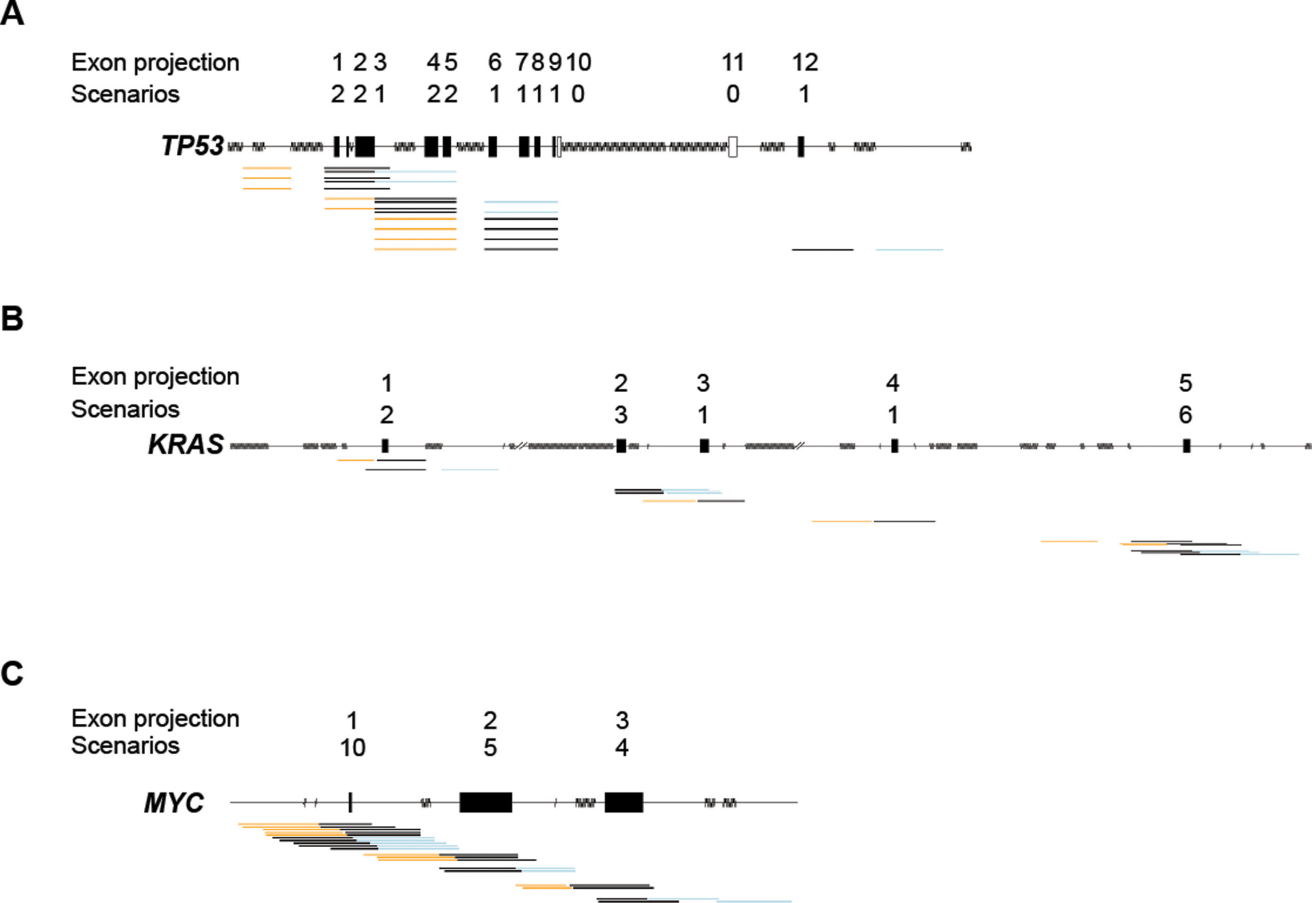
Knock-in construct designs to the cancer genes *TP53, KRAS* and *MYC*. Graphical output of potential knock-in scenarios for exon projections of *TP53* (A), *KRAS* (B) and *MYC* (C) with number of scenarios per exon projection. The exon projections are represented by boxes, solid (with scenarios) or empty (without scenarios); regions with repeat sequences by a string of vertical bars; potential homology arms by horizontal lines in orange (left HA), light blue (right HA) and black (HA spanning an exon).

We attempted to design at least one rAAV knock-in strategy to introduce mutations in each of the exons or exon projections, aiming to suggest additional scenarios for each exon and rank the alternatives by several empirical criteria known to affect targeting efficiency (Figure 2). First, we identified at least one HA arm spanning the whole exon of interest and tried to match it with an upstream or downstream homology arm to create two principal groups of scenarios—left-span and span-right. When more than one HA existed in each category (span, left or right) we performed a cluster analysis of the HA collection using the DBSCAN algorithm. The clustering of highly similar HAs reduced the complexity of matching HA for alternative scenarios. Each arm from a span cluster was matched to each arm from the associated right or left clusters, for ∼9.38 × 10^11^ possible HA pair-matches. By restricting the HA pair matches to those generating a gap <700 bases, not having exons in the gap and not ending in exons the complexity was reduced 5-fold to ∼1.76 × 10^11^ possible designs. Next, only the best match having the smallest gap size between homology arms and the smallest distance from the split point to the exon-intron borders, a total of ∼3.50 × 10^7^ (5000-fold reduction) possible scenarios, was evaluated further. The best matches in each of these cluster-cluster attempts represent alternative categories of gene knock-in scenarios. If more than five alternative scenarios were available for each left-span or span-right design, we ranked these scenarios (see Materials and Methods) and saved the five best, thereby limiting the output to 10 scenarios per exon. The criteria to score and rank the best cluster-cluster HA matches included priority over smaller gap, smaller distance from the split-point to the exon-intron border of the span arm and longer cumulative HA length. Scenarios sharing an HA or highly similar HAs (differing by no more than 5 bases in each end) were grouped as an undirected graph and only the best scoring in each graph was kept. Grouping of similar scenarios and overall scoring reduced the available choices for final evaluation by humans by ∼98% and presented maximum 10 alternative knock-in scenarios per exon. By this approach, we were able to design 909 500 knock-in scenarios for 154 377 protein-coding exons or exon projections from 17 559 GeneIDs, making 23 165 270 bases in exons accessible for knock-in by the rAAV technology (Figure 2, Table 1 and Supplementary Table S3). Graphical representations of the output for the cancer genes *TP53, KRAS* and *MYC* are presented in Figure 3. To assess the amplification efficiency of all suggested knock-in designs for *TP53, KRAS* and *MYC*, we performed PCR reactions with the primer pairs for each homology arm (Supplementary Figure S1). We found that the amplification efficiency was 100%, for *TP53, KRAS* and *MYC* genes.

**Table 1.** Knock-in designs to the CCDS transcriptome

Chr	Genes	Genes with knock-in scenario	CCDS exons or exon projections	CCDS exons with knock-in scenario	Mean scenarios per covered gene	Mean scenarios per covered exon	CCDS bases covered (%)
1	1936	1836	19 266	15 963	51.37	5.91	74.70
2	1173	1131	14 440	12 173	62.60	5.82	73.45
3	1019	972	11 242	9389	57.33	5.93	74.52
4	723	668	7480	6159	49.58	5.38	69.48
5	833	785	8599	7012	49.89	5.59	67.61
6	986	944	9452	8103	50.61	5.90	74.94
7	845	794	8689	7098	52.29	5.85	70.07
8	631	608	6296	5281	49.64	5.72	74.18
9	735	683	7709	6392	56.54	6.04	71.72
10	709	681	7822	6485	55.66	5.85	73.54
11	1209	1140	10 499	8974	48.37	6.15	76.41
12	978	932	10 585	8569	53.44	5.81	74.92
13	307	292	3336	2812	54.03	5.61	70.17
14	576	537	5734	4680	51.69	5.93	70.68
15	555	532	6842	5696	63.86	5.96	72.99
16	780	746	8077	6191	51.27	6.18	68.43
17	1099	1063	11 251	9283	54.83	6.28	75.82
18	260	247	2919	2411	53.96	5.53	71.61
19	1343	1145	11 027	7383	36.05	5.59	50.27
20	516	491	4716	3694	45.65	6.07	68.55
21	207	197	1943	1666	52.09	6.16	79.43
22	411	387	3956	3189	51.53	6.25	69.37
X	791	708	6610	5446	45.51	5.92	69.09
Y	45	40	410	328	48.20	5.88	68.85
**Total**	**18667**	**17559**	**188 900**	**154 377**	**51.80**	**5.89**	**71.20**

### Generation of knock-out scenarios for protein-encoding genes

The established strategy to achieve a gene knock-out is to introduce an alteration in the genome causing production of a frameshifted or prematurely truncated mRNA transcript leading to non-sense-mediated mRNA decay or synthesis of a defunct protein. An alternative approach is to excise an entire exon or a part of an exon to obtain a frameshift or introduce a premature stop codon. We therefore sought all suitable designs for deletion-based knock-out strategies. A necessary condition for gene knock-out is that the targeted exon needs to be present in all alternative transcript forms. Out of 305 464 CCDS exons from 18 667 genes there were 165 169 (∼54%) common exons from 18 424 genes (∼99%), 109 288 exons present in 12 594 genes with only one transcript form and 55 881 exons from 5830 genes present in all alternative transcript forms. A total of 97 238 (∼32%) exons from 15 487 genes (∼83%) were present in all transcript forms and had a length not divisible by three; these exons may therefore be suitable targets for knock-out designs. We attempted whole exon deletion designs only if the exon length was ≤700 bases. Of the total ∼1.26 × 10^11^ scenarios screened, ∼3.47 × 10^10^ fulfilled our inclusion criteria for gap size limit and ∼5.96 × 10^6^ were selected as the best in the cluster-cluster matching (∼6000-fold reduction). Finally, we found 157 746 gene knock-out scenarios for 50 664 exons (∼52.1% of suitable targets) from 13 443 genes (∼86.8% from suitable targets) (Table 2 and Supplementary Table S4). In principle, many knock-in scenarios can be used for introduction of one or several premature stop codons in a desired exon. Thus, the gene knock-in database can also be used to generate gene knock-outs. Such a complementary design was available for 79 267 (∼81.5%) of the exons eligible for gene knock-out.

**Table 2.** Deletion knock-out designs to the CCDS transcriptome

Chr	Genes with a shared exon of length non divisible by 3	Genes with ≥1 knock-out scenario	Shared CCDS exons of length non divisible by 3	CCDS exons with knock-out scenario	Mean scenarios per targeted gene	Mean scenarios per targeted exon
1	1578	1366	9853	5066	11.66	3.14
2	1039	933	7109	3834	12.45	3.03
3	857	759	5801	3068	12.57	3.11
4	597	551	3974	2087	11.54	3.05
5	698	599	4670	2343	11.71	2.99
6	789	701	4726	2638	11.93	3.17
7	699	603	4407	2333	12.09	3.13
8	548	490	3309	1718	10.77	3.07
9	599	527	3846	2091	12.61	3.18
10	612	546	3917	2099	11.93	3.10
11	886	770	5386	3040	12.25	3.10
12	839	716	5381	2690	11.62	3.09
13	259	239	1664	907	11.24	2.96
14	453	404	2957	1549	12.00	3.13
15	485	436	3622	1939	14.17	3.19
16	693	584	4278	2161	11.74	3.17
17	957	841	5998	3272	12.33	3.17
18	215	194	1460	740	11.40	2.99
19	1152	859	5783	2355	8.63	3.15
20	436	369	2513	1253	10.49	3.09
21	135	117	949	509	13.68	3.15
22	365	310	2163	1134	11.45	3.13
X	564	501	3272	1720	11.01	3.21
Y	32	28	200	118	12.32	2.92
**Total**	**15 487**	**13 443**	**97 238**	**50 664**	**11.73**	**3.11**

### Development of a recombination-based vector system for rAAV generation

**Figure 4.**
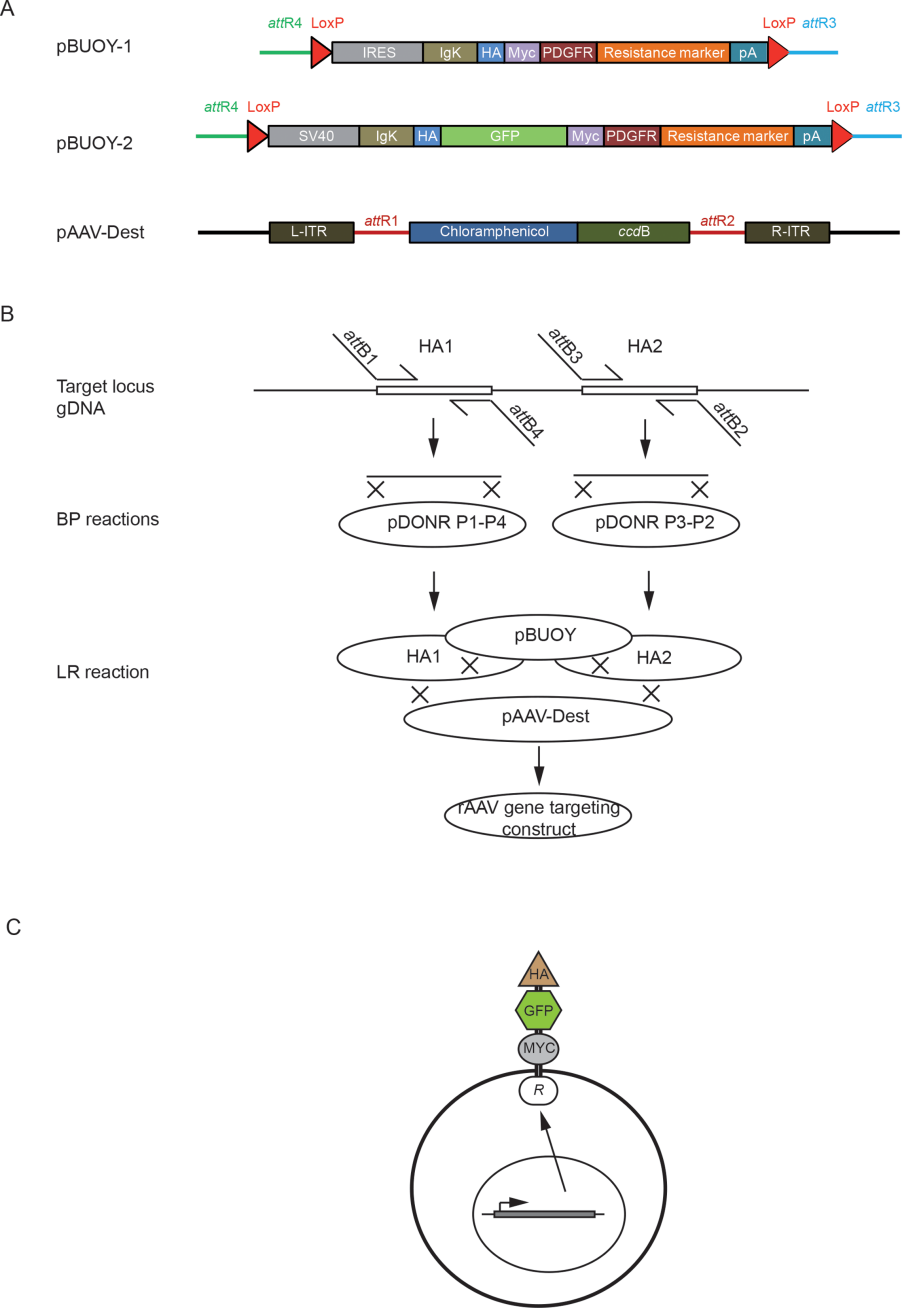
A recombination-based vector system for gene targeting with sortable cell surface markers. (**A**) The pBUOY vector family, encoding fusion genes having extracellular epitopes and intracellular resistance genes, was developed. A destination vector, pAAV-Dest, was created to provide AAV2 LTRs to the recombination product. (**B**) Construction strategy for gene targeting vectors. Homology arms (HA1 or HA2) of 0.8–1.1 kb are PCR amplified from the target locus using att-flanked primers. Second, the homology arms are recombined into pDONR vectors in Gateway BP reactions. Third, homology arms in pDONR plasmids are recombined with a transmembrane fusion gene encoding extracellular murine Ig K-chain leader (IgK), hemagglutinin A (HA), GFP and Myc epitopes, a transmembrane domain of the PDGFR β-receptor (PDGFR tm) and intracellular drug resistance activity and the destination pAAV vector in a four-way Gateway LR reaction. (**C**) Targeted cells express a fusion resistance gene for selection or sorting using HA-A or Myc epitope antibodies.

To enable rapid and automatable generation of rAAV constructs we designed a Gateway compatible rAAV vector system (Figure 4, Supplementary Figure S2). The approach encompasses (i) PCR amplification of homology arms using primers tailed with an appropriate *att* recombination site, (ii) recombination of the homology arm PCR products into pDONR^TM^ Px-Py entry vectors in Gateway BP reactions, (iii) directional recombination of homology arm vectors and a vector encoding a promoter-driven selection markers fused to extracellular GFP and cell sorting epitopes into a destination vector containing the AAV ITRs in a Gateway LR reaction and (iv) identification of correctly assembled constructs by PCR. Sorting vectors with five different selection markers (blasticidin, hygromycin, neomycin, puromycin and zeomycin) were designed to provide the ability to target multiple alleles without removing the selection cassette from the already targeted allele (Supplementary Figure S2). To evaluate strategies to obtain an increased fraction of resistant clones with desired integrations, we engineered (i) promoterless IRES-containing resistance gene fusion constructs, (ii) promoter-containing resistance gene fusion constructs and (iii) a promoter containing construct with FMDV2A self-cleavable peptide and splice donor site but lacking the polyA tail.

### Generation of rAAV constructs by Gateway recombination

**Figure 5.**
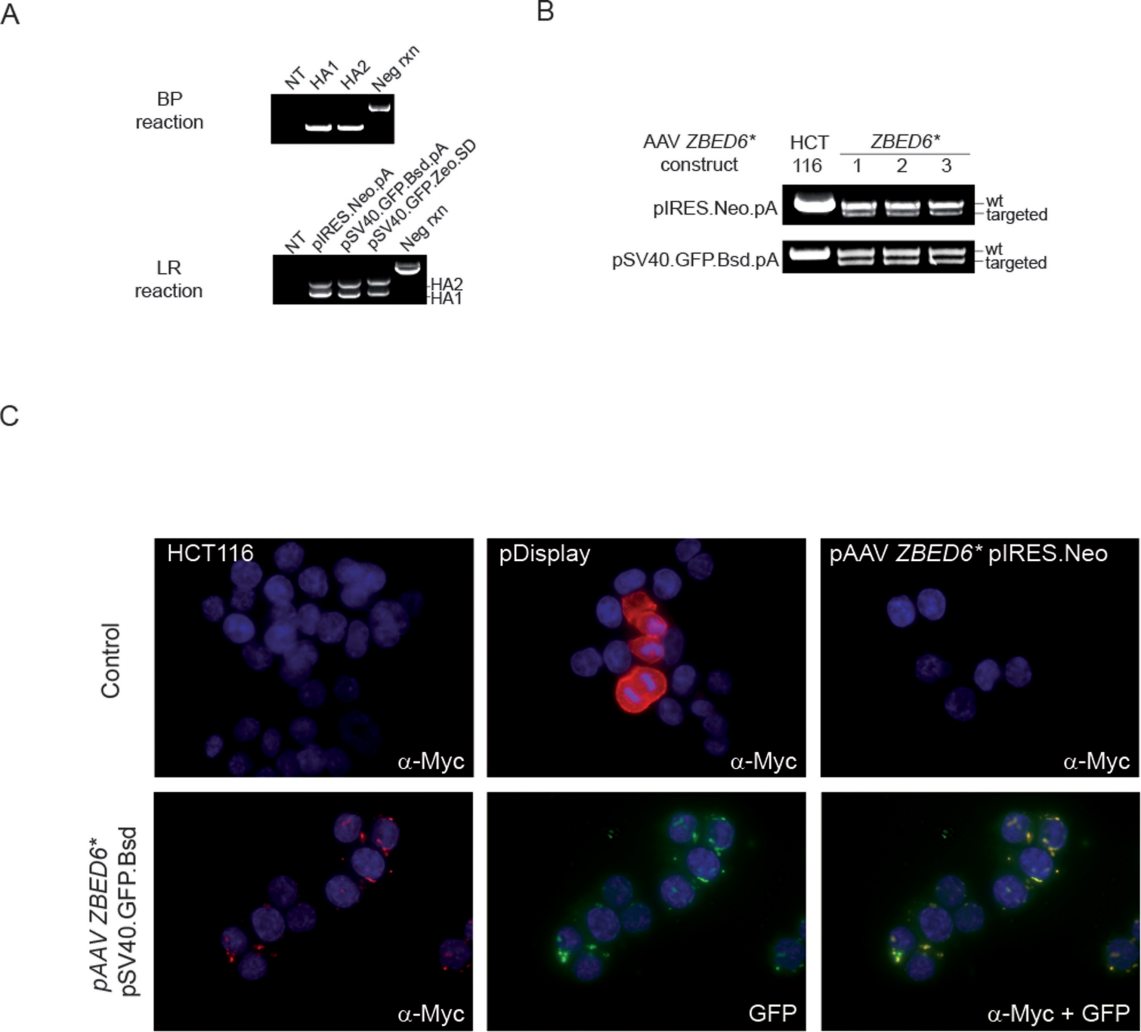
Recombination-based generation of rAAV targeting constructs with a transmembrane selection and sorting marker and targeting in human cancer cells. (**A**) Locus-specific homology arm sequences from the *ZBED6* gene were recombined with selection marker and pAAV packaging sequences using the MultiSite Gateway system. Upper panel, colony PCRs of no template (NT), empty destination vector (control, 2.5 kb) and entry clones from BP recombination reactions for ZBED6* homology arms using locus independent universal primers. Lower panel, colony PCRs of LR reactions of desired recombination products for ZBED6* pIRES.Neo and pSV40.GFP.Bsd constructs and empty destination vector (control, 1.9 kb). (**B**) PCR detection in three different cell clones per construct of ZBED6* pIRES.Neo and pSV40.GFP.Bsd integration in the target locus after selection. (**C**) Expression of transmembrane selection markers in HCT116 colorectal cancer cells. Myc immunofluorescence was observed in transient transfection of pDisplay (positive control) and in cells targeted with promoter-driven ZBED6* pSV40.GFP.Bsd but not in untransfected cells (HCT116) or in cells targeted with the ZBED6* pIRES.Neo promoter-less construct. GFP signals from ZBED6* pSV40.GFP.Bsd co-localize with Myc signals (lower right panels).

Homology arms for five different gene targeting constructs were PCR amplified, tailed with recombination directing sequences and recombined into MultiSite Gateway Entry vectors. The BP reaction consistently yielded >1000 kanamycin resistant colonies when one-fifth of the 10 μl reaction was used in transformations. The success rate of GateWay BP reactions was 100% based on the 10 different homology arms attempted (Figure 5A and data not shown). Construct assembly by 4-way Gateway LR reactions yielded 10–1000 clones in a homology arm-dependent manner based on five different attempted constructs (Figure 5B and data not shown). To achieve locus-independent screening for desired BP and LR reaction products, we devised a homology arm-independent PCR amplification strategy. This strategy provided an internal positive control for the PCR reaction and a negative control for the insert by amplifying a PCR product of 2519 bp from empty pDONR P1-P2 or P3-P2 vectors in case of BP reaction and 1925 bp from the empty destination vector (Figure 5A).

### Expression of transmembrane marker in target cells

Promoterless and promoter-containing gene targeting constructs, both with identical homology arms, targeting the transcription factor *ZBED6*, were packaged into rAVV particles by co-transfection with pHelper and pAAV.RC plasmids into the AAV293 packaging cell line. Crude cell lysates containing rAVV particles were harvested and used to infect HCT116 colorectal cancer cells. After 24 h of infection and 2 weeks of selection, clones with desired integrations were identified by PCR (Figure 5B). To generate gene targeting constructs that contain epitope-tagged resistance gene we have taken an advantage of pDiplay vector that contained the HA-A and MYC epitopes. A GFP tag was subsequently cloned into the second generation of the vector to facilitate fluorescence-activated cell sorting (FACS)-based cell sorting. In addition to HCT116 cells transiently transfected with pDisplay, the cell clones derived from promoter containing constructs after selection showed cell membrane associated Myc-staining that also co-localized with GFP signals (Figure 5C). In contrast, the promoterless IRES-containing construct, depending on the endogenous promoter for expression, did not give rise to Myc-expressing cell clones despite conferring resistance to the antibiotic selection.

## DISCUSSION

Genome editing of human somatic cells is rapidly becoming integral to understanding gene function. Whereas gene knock-out is a one step process, and thus more efficient, with Cas9/CRISPR systems and ZFN-based approaches, all genes are not amenable to such targeting and off-target integrations may be challenging. Even in the era of highly efficient and customizable molecular scissors, true isogenic cell models are not easily achievable due to off-target mutagenesis. Whereas in case of knock-outs, not all the knock-out clones generated in the same experiment using the same molecular scissor, are isogenic because the small deletions are generated through NHEJ repair of DSBs at the target site and are not of same size (15). On the other hand, rAAV technology solely relies on HR-based insertion of gene targeting construct and results in a highly defined alteration throughout the clones. For knock-in strategies, especially to characterize somatic point mutations observed in cancer genomes, rAAV offers the benefit of specific integrations with very little off-target effects. rAAV-mediated genome editing does not introduce off-target DSBs in contrast to ZNF-based (5) or Cas9/CRISPR systems (27). However, random integration events have been reported (18) albeit less than in the comparable gene targeting techniques (18,28). A rough estimate based on the published literature suggest that for rAAV-mediated technology ∼3% of the successfully targeted clones may have an accompanying random integration event (18). Next-generation sequencing analyses revealed no random integration events in mitochondrial genomes after rAAV-mediated gene transfer (29), however, the random integration in the genome is a concern in therapy applications (30). Gene knock-out by rAAV is more challenging and will likely only be used to target genes where no other approach is available. For all editing approaches, the complexity of the transcriptome creates a challenge when a desired alteration in one transcript variant affects another transcript form of the same gene. Many human genes give rise to multiple transcript variants, produced by alternative transcription initiation, termination or splicing. It is known that ∼95% of human multi-exon genes undergo alternative splicing (31) and ∼50% of human multi-transcript genes use alternative promoters (32). We therefore superimposed the genomic coordinates of the exons of all transcript variants to create new features termed exon projections to manage potential problems in knock-in scenarios. All alternative CCDS transcripts would therefore safely be targeted by the designs presented here; the mean and median length of the exon projections is higher than that of the native exons because exon projections are never smaller than the individual projected exons. From previous studies, the length of a homology arm for rAAV editing can be in the range 700–1200 bp; this range was therefore chosen as the desired PCR product size range. Since the cumulative length of the homology arms can influence the targeting efficiency, the computational algorithm was designed to prefer longer arms if possible for maximizing the targeting efficiency (Supplementary Figure S3). The average target sequence, an exon projection with 3 kb flanking sequences, was 6.17 kb. We chose a SW approach to force primer design also in regions which may otherwise be down-prioritized by primer design software and ensure good sequence coverage by generation of overlapping PCR products. The SW should be smaller than the targeted sequence region, bigger than the maximum HA lengths, and smaller than two maximum homology arm lengths to allow freedom for optimal primer design. In practice, the theoretical maximum sequence coverage is not an attainable goal, since primer design in certain regions does not give suitable primer pair candidates. During optimization of HA generation the coverage of the target sequence was approaching a maximum at 66.5%. To achieve such total sequence coverage, with an average of >2000 different HAs spanning a targeted sequence position and to finish in a reasonable time-frame, we selected the SS value of 50 and and NPP value of 50. From all combinations of NPP and SS with comparable projected computational time and similar sequence coverage parameters, this combination had the best average sequence coverage per average penalty value, an indicator not only of the mean sequence coverage but also for the quality of the primer pairs.

In our effort to find gene knock-in scenarios, one or more designs were suggested for 81.7% of the exon projections of the CCDS exons, covering 71.2% of protein-coding base positions. In the process, ∼7.09 × 10^8^ homology arms were generated and assessed. Although it would be possible to generate additional HAs, the process is currently not limited by the availability of HAs as 98.4% of exons in 99.9% of CCDS genes in the exon projection database had available HAs. However, we were not able to suggest knock-in scenarios for all protein-coding genome positions when the construct design criteria were applied; in particular, repetitive sequence regions proved a major reason for design failure. Nine percent of exons or exon projections bordered 1 kb of >75% repeat sequences and 0.5% were flanked by such sequence regions on both sides. For gene knock-out, at least one design for 52.1% of exons for 86.8% of the genes was suggested under the requirements that the targeted exon (i) is present in all known alternative splice variants and (ii) the targeted exon length was non-divisible by 3. Homology arms were present for 96% of the knock-out suitable exons for 99.8% of the genes. Covering all protein-coding exons is desirable from theoretical point of view, however, to achieve it in practice different design criteria need to be violated in every specific case. In the gene knock-out approach by exon deletion we were not able to suggest scenarios for ∼48% of the exons. Although HAs were available for many of these exons, a complete knock-in or knock-out scenario was impossible while adhering to the design criteria. A possible approach for genes were knock-out scenarios are not available is to use a knock-in scenario to introduce stop codon in a desired exon. For 30 269 (∼65%) of the exons defined as eligible for knock-out but without a suggested design there was a complementary knock-in scenario available. Together with the 50 664 exons with knock-out designs there was at least one option available for 80 933 (∼83.2%) of all exons eligible for knock-out or potentially available designs for 15 057 protein coding genes (∼80.7%). This compares favorably to technologies such as Cas9/CRISPR that can access ∼40.5% of human exons (11). However, one appropriate knock-out scenario is enough to disable gene function and not all exons of a gene would be considered for targeting. For example, knock-out designs to the first or last exon of a gene are typically less interesting, as well as exons not encoding functional domains; user input is therefore necessary when choosing the final knock-out scenario. Targeting exons close to the 3′-end of the gene in knock-out scenarios may be less effective for disruption of protein function and generally not recommended (33). On average, we suggest 5.9 knock-in and 3.1 deletion knock-out scenarios per exon. There are several explanations why the number of scenarios per exon in knock-out strategies is smaller. First, we limited the knock-out possibilities to 10 for each exon. Second, exons have a median length of 122 bases which reduces the possibility to place HAs within the exon borders in knock-out designs. Third, a whole exon deletion is proposed only if an exon is less than 700 bases, which excludes a small fraction of exons. The chance to find knock-out scenarios with a small gap between the HA decreases with increasing size of the targeted exon. Contrary to gene knock-outs, the short median exon length and independence of gap size between the HAs facilitates knock-in designs. An increased maximum gap size would result in more designs, at the expense of targeting efficiency. Scenarios with small or no gap between the homology arms were prioritized to minimize sequence deletions as a result of the genome editing. However, we did not exclude designs generating larger gaps, as for some genomic regions with high complexity better scenarios were not available. The final selection of targeting construct design may be guided by user preferences or amplification efficiency of homology arm primers. It is therefore suggested that the amplification efficiency of the homology arms in the different designs is evaluated by PCR to select the most efficient one.

Gene editing by rAAV-assisted HR has been hampered by extensive cloning and cell culture expansion work, resulting in a turnaround of 3–12 months. We here also describe a Gateway compatible vector system for the construction of AAV gene targeting vectors, which is rapid, efficient and potentially automatable. Gateway cloning offers a major advantage to conventional cloning in that the recombination reaction is independent of the target sequence. As restriction sites are frequently present in the homology arm sequences, this removes a major design constraint in gene targeting using rAAV. Further, the recombination reaction can be automated and its uniformity has made it a technology of choice in large-scale projects. The universal screening primers for BP and LR products presented here provide a convenient way to screen the outcome of many different recombination reactions in parallel, as homology arm sizes are reflected in PCR product length and failed reactions lacking homology arms yield products of specific sizes. Sorting strategies based on promoterless gene targeting constructs, such as the IRES-containing pSEPT vector (26), can be envisioned. Recent improvements have enabled FACS-based enrichment of cells with promoterless rAAV integration in a highly expressed gene (*CENP-A*), but successful targeting and enrichment has not been demonstrated for other genes (34). However, we did not succeed at reliably obtaining fluorescence signals of enough intensity to enable FACS sorting using such constructs (Figure 5C and data not shown).

The field of gene targeting is quickly evolving and there are many competing technologies available. One of the technologies currently in use is the Cas9/CRISPR system, which can give up to 68% targeting efficiency in variety of cell lines and has the advantage of facile multiplexing and easy customization (35). Although Cas9/CRISPR systems are a current preferred choice for bi-allelic gene knock-outs, we see a merit of rAAV-mediated gene editing for applications related to knock-in of point mutations or large gene transfers. For example, rAAV-based technologies are the primary choice for the CRISPR/Cas9 delivery in cells and organisms (36,37). A niche application where rAAV technology has advantage over any NHEJ-based gene targeting is the correction of mutations in mononucleotide repeats in mismatch repair-deficient colorectal cell lines (our unpublished results). On a more hypothetical basis, it also has merit if the desired target gene modification is incompatible with the other available gene targeting techniques.

This work focused on the protein-encoding exons of the genome. We attempted to design scenarios for editing in ∼1.05% of the human genome sequence and suggested knock-in scenarios for ∼0.75% of positions of the human genome. However, the algorithms and vectors can easily be adapted to target additional elements of the genome such as RNA genes, promoters, transcription factor binding sites and elements defined by the ENCODE consortium (38). The ENCODE project defines a biochemical function to ∼80.4% of the sequence of the human genome. If we extrapolate our result to this target sequence, this translates to ∼1.77 × 10^9^ potentially rAAV-accessible genome positions.

## SUPPLEMENTARY DATA

Supplementary Data are available at NAR Online.

SUPPLEMENTARY DATA
