# Supplementary material for: Computational and molecular tools for scalable rAAV-mediated genome editing
Source: Nucleic Acids Res. 2014 Dec 8;43(5):e30. doi: 10.1093/nar/gku1286 (PMC4357690; doi:10.1093/nar/gku1286)
Supplement: SUPPLEMENTARY DATA [file supp_43_5_e30__index.html]

Computational and molecular tools for scalable rAAV-mediated genome editing — SUPPLEMENTARY DATA 

# Computational and molecular tools for scalable rAAV-mediated genome editing

## SUPPLEMENTARY DATA

**Files in this Data Supplement:**

- SUPPLEMENTARY DATA
- SUPPLEMENTARY DATA
- SUPPLEMENTARY DATA
- SUPPLEMENTARY DATA
