# Supplementary material for: Computational and molecular tools for scalable rAAV-mediated genome editing
Source: Nucleic Acids Res. 2014 Dec 8;43(5):e30. doi: 10.1093/nar/gku1286 (PMC4357690; doi:10.1093/nar/gku1286)
Supplement: SUPPLEMENTARY DATA [file supp_gku1286_nar-02914-met-h-2014-File007.pdf]

Supplementary Figure 1. PCR amplification of suggested homology arms for knock-in construct designs to the cancer genes *TP53*, *KRAS* and *MYC*. Each pair of columns represents left (L) and right (R) homology arm of the knock-in designs for *TP53* (A), *KRAS* (B) and *MYC* (C). Designs in ascending order of the genomic start coordinate of the left homology arm. The numbers above each design represent the targeted exon projection in respective gene. D. Summary table for the amplification efficiency of the homology arms and knock-in designs for *TP53*, *KRAS* and *MYC* genes.

Supplementary Figure 2. Vector family for recombination-based assembly of gene targeting constructs with extracellular sorting tags. A. Transmembrane fusion genes encoding extracellular HA, EGFP, and Myc epitopes and intracellular markers conferring resistance to puromycin, blasticidine, hygromycin, zeocin and neomycin were engineered and flanked by *att*B4r and *att*B3r sites (green) for use in four-way Gateway recombination and *loxP* sites (yellow) to allow removal of the resistance cassette. SV40 promoter; Sp, signal peptide; HA, hemagglutinin epitope; EGFP, enhanced green fluorescent protein; Myc, c-Myc epitope; PDGFR tm, transmembrane domain of the PDGF β-receptor; pac, puromycin N-acetyl-transferase; bsd, blasticidin S deaminase; hph, hygromycin B phosphotransferase; Sh ble, s. hindustanus bleomycin gene; aph, aminoglycoside 3' phosphotransferase. B. Destination vector pAAV-DEST.

Supplementary Figure 3. Distribution of the cumulative length of the homology arms for all suggested gene knock-in and knock-out designs. The graph represents the total number of designs for each cumulative length present in the database of gene knock-in (A) or gene knock-out (B) scenarios.

Supplementary Figure 1

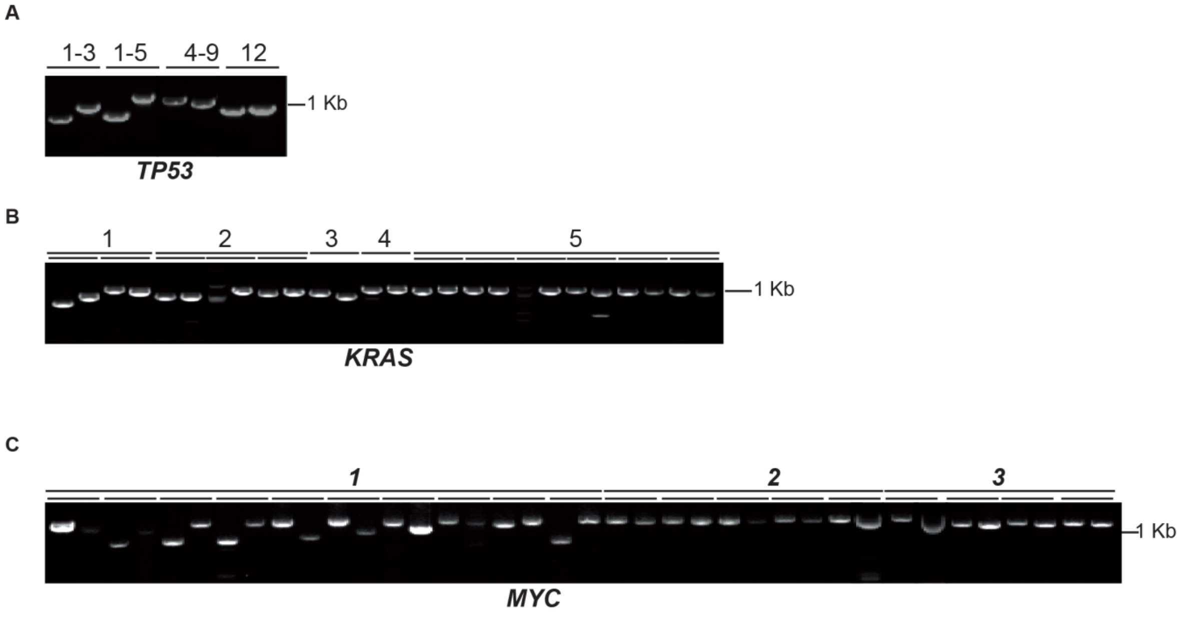

D

Target gene	Successful/Attempted HAs	Successful/Attempted Designs
TP53	8/8	4/4
KRAS	26/26	13/13
MYC	38/38	19/19
All three genes	72/72	36/36

Supplementary Figure 2

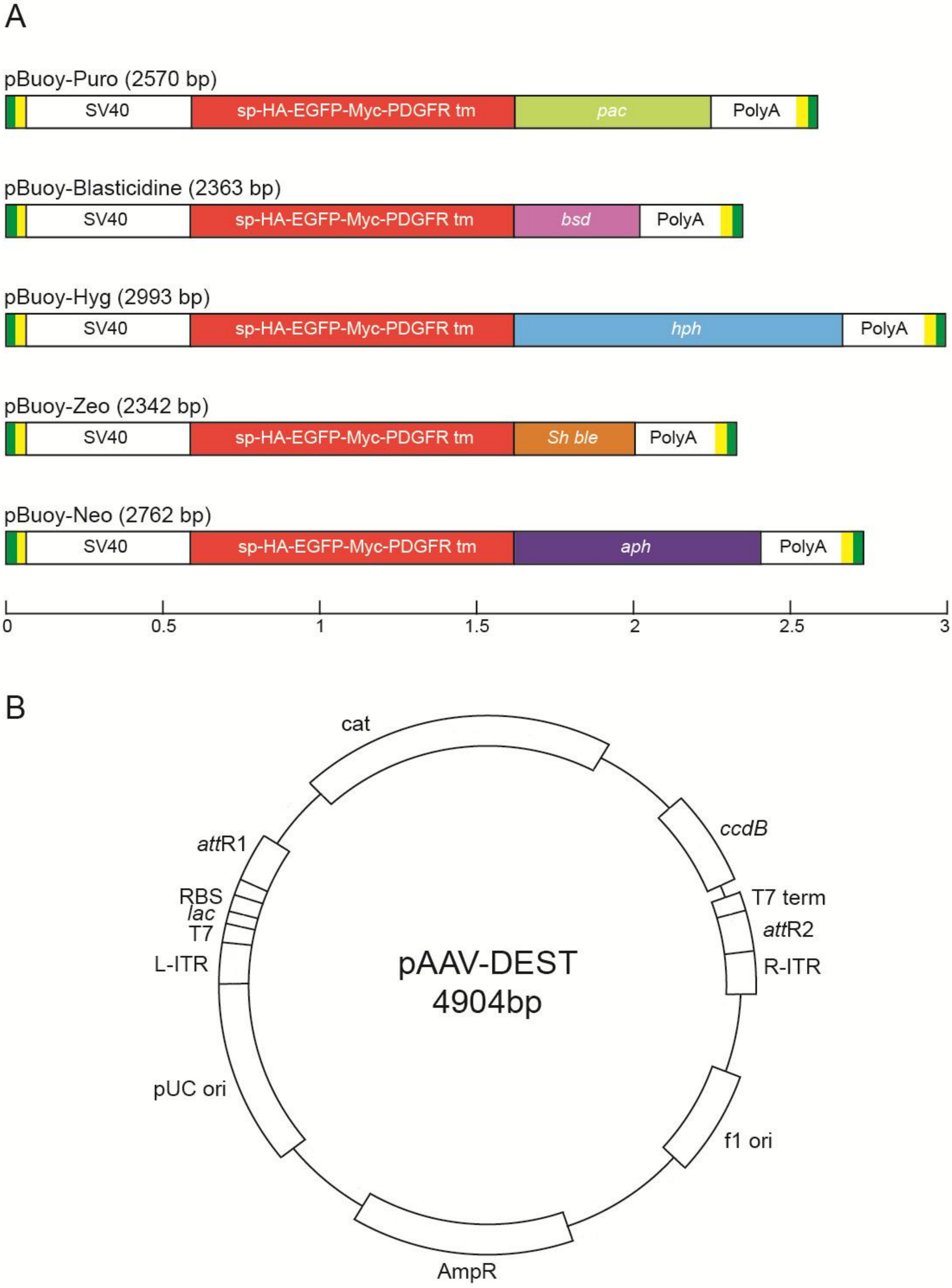

A

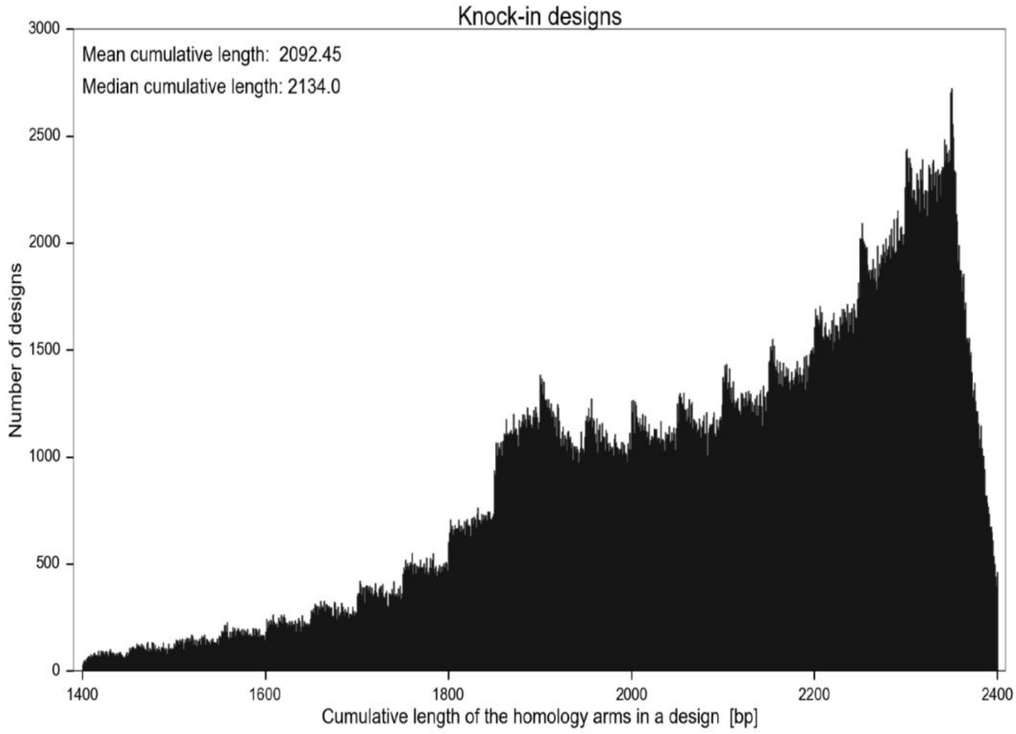
Knock-in designs
Mean cumulative length: 2092.45
Median cumulative length: 2134.0
Number of designs
0
500
1000
1500
2000
2500
3000
1400
1600
1800
2000
2200
2400
Cumulative length of the homology arms in a design [bp]

B

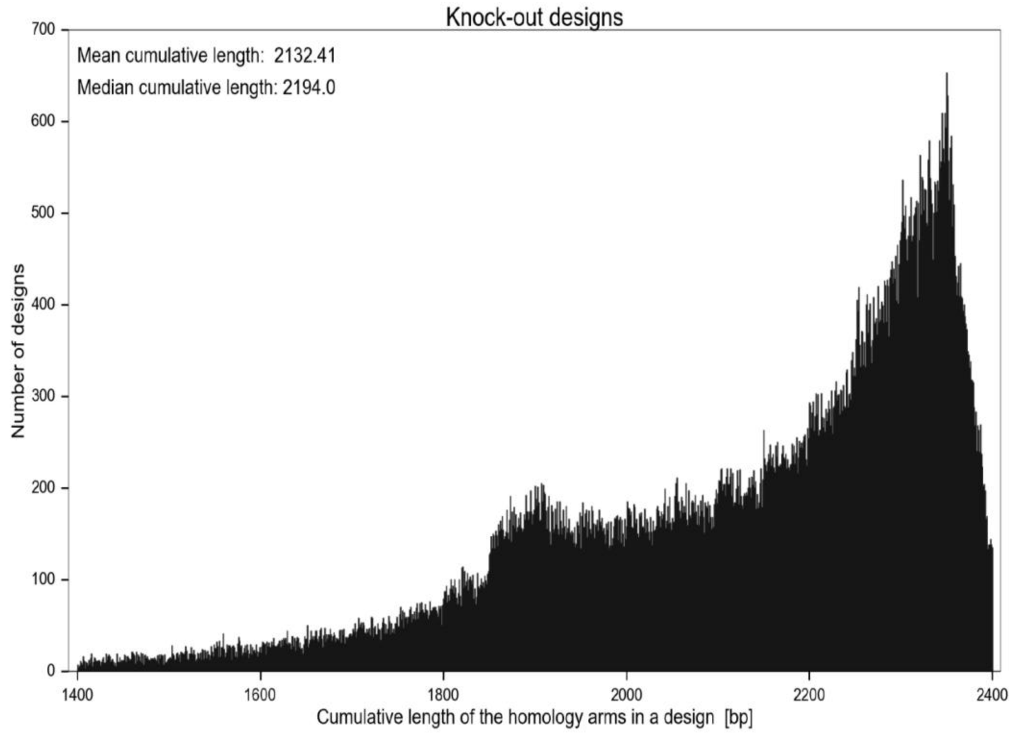
Knock-out designs
Mean cumulative length: 2132.41
Median cumulative length: 2194.0
Number of designs
0
100
200
300
400
500
600
700
1400
1600
1800
2000
2200
2400
Cumulative length of the homology arms in a design [bp]

Supplementary Table 1. Oligonucleotide primer pairs for PCR

ID	PRIMER NAME	PRIMER SEQUENCE
1		ATAAGAATGCGGCCGCTAATACGACTCACTATAGGGG
2		CCTGGGGTGCCTAATGCCGCCATAGTGACTGG
3		AGTCACTATGGCGGCATTAGGCACCCCAGGCTTTAC
4		ATAGTTTAGCGGCCGCCAATCCGGATATAGTTC
5	T_1_F	GGGACAACTTTTCTATACAAAGTTGATAACTTCG
6	T_1_R	TGTGTCTGTCTCCATGGTTGTGGCAAGCTTATCATC
7	T_2_F	AAGCTTGCCACAACCATGGAGACAGACACACTCCTG
8	T_2_R	TTCAATGGCCGATCCACGTGGCTTCTTCTGCCAAAG
9	T_3_F	CAGAAGAAGCCACGTGGATCGGCCATTGAACAAG
10	T_3_R	GGGACAACTTTATTATACAAAGTTGTATAACTTCGTATAATG
11	G_1F	TCAGGACTGCAGCAAGGGCGAGGAGCTGTT
12	G_1R	TCAGGACTGCAGCTTGTACAGCTCGTCCATGC
13	LOXPIRES F	ATAACTTCGTATAGCATACATTATACGAAGTTATCCGCCCCTCTCCC TAACGTTACTGGCCGAAGC
14	IKGIRES R	TGTGTCTGTCTCCATGGTTGTGGCCATATT
15	IRESIKG F	AATATGGCCACAACCATGGAGACAGACACACTCCTG
16	BSDPDGF R	TTGAGACAAAGGAACACGTGGCTTCTTCTGCCAAAG
17	HYGROPDGF R	GGTGAGTTCAGGCTTTTTACGTGGCTTCTTCTGCCAAAG
18	NEOPDGF R	AATCCATCTTGTTCAGCACGTGGCTTCTTCTGCCAAAG
19	PUROPDGF R	GCTTGTACTCGGTAACACGTGGCTTCTTCTGCCAAAG
20	ZEOPDGFR R	GCACTGGTCAACTTGGCACGTGGCTTCTTCTGCCAAAG
21	PDGFBSD F	CAGAAGAAGCCACGTGTTCCTTTGTCTCA
22	PDGFBSD R	CAGAGTCCCGCTCAGCCCTCCCACACATAACCAGA
23	PDGFHYGRO F	CAGAAGAAGCCACGTAAAAAGCCTGAACTCACC
24	PDGFHYGRO R	CAGAGTCCCGCTCATTCCTTTGCCCTCGGACGAG
25	PSEPT-2R	AATCCATCTTGTTCAGCGGATCGGCCATTGAACAAG
26	PSEPT-3F	CAGAAGAAGCCACGTGCTGAACAAGATGGATT
27	PUR2_3F	CAGAAGAAGCCACGTGTTACCGAGTACAAGC
28	PUR2_3R	CAGAGTCCCGCTCAGGCACCGGGCTTGCGGGTCA
29	PDGFZEO F	CAGAAGAAGCCACGTGCCAAGTTGACCAGTGC
30	PDGFZEO R	CAGAGTCCCGCTCAGTCCTGCTCCTCGGCCACGAAGTG
31	BSDPA F	TCTGGTTATGTGTGGGAGGGCTGAGCGGGACTCTG
32	HYGROPA F	CTCGTCCGAGGGCAAAGGAATGAGCGGGACTCTG
33	PUROTGAPA F	TGACCCGCAAGCCCGGTGCCTGAGCGGGACTCTG
34	ZEOPA F	CACTTCGTGGCCGAGGAGCAGGACTGAGCGGGACTCTG
35	LOXPSV40 F	CAAAGTTGATAACTTCGTATAGCATACATTATACGAAGTTATTAGGTC TGAA
36	SV40 R	CAGGAGTGTGTCTGTCTCCATGGTGGCTCTAGCCTTA
37	SV40 IKG F	AAGGCTAGAGCCACCATGGAGACAGACACACTCCTG
38	Bsd FMDV2A SD F	CTGGTTATGTGTGGGAGGGCAATTTTGACCTTCTCAAGTTGG
39	Hygro FMDV2A SD F	CTCGTCCGAGGGCAAAGGAAAATTTTGACCTTCTCAAGTTGG
40	Neo FMDV2A SD F	GCCTTCTTGACGAGTTCTTCAATTTTGACCTTCTCAAGTTGG
41	Puro FMDV2A SD F	TGACCCGCAAGCCCGGTGCCAATTTTGACCTTCTCAAGTTGG
42	Zeo FMDV2A SD F	TCGTGGCCGAGGAGCAGGACAATTTTGACCTTCTCAAGTTGG
43	FMDV2A SD 1R	TGCTATACGAAGTTATCAACGAAGTTCCT
44	FMDV2A SD 2R	gttgtATAACTTCGTATAATGTATGCTATACGAAGTTAT
45	M13F	GTAAAACGACGGCCAG
46	M13R	CAGGAAACAGCTATGAC
47	DV1LRSCR1 F	GGCCGCTAATACGACTCACT
48	AAVSV40 R	GCTTGGCTGGACGTAAACTC
49	PALRSCR3 F	CGGGATGAGTTGGGAATAAC
50	DV1LRSCR4 R	CTTTCGGGCTTTGTTAGCAG
